# Setting the stage for strengthened annual monitoring of family planning program performance at the state/national level in Myanmar

**DOI:** 10.12688/gatesopenres.13012.2

**Published:** 2019-12-10

**Authors:** Khaing Nwe Tin, Jessica Williamson, Emily Sonneveldt

**Affiliations:** 1 Department of Public Health, Ministry of Health and Sports, Myanmar, Nay Pyi Taw, 15011, Myanmar; 2 Track20, Avenir Health, Washington DC, USA; 3 Track20, Avenir Health, Connecticut, USA

**Keywords:** Family Planning, monitoring, Myanmar

## Abstract

**Background:** Although Myanmar has made good progress in family planning by increased contraceptive prevalence rate (CPR) from 41% in 2007 to 52.2% in 2016, it remains lower than the target of 60% by 2020. There are also huge disparities sub-nationally, ranging from 25% to 60%. While there is a strong need to monitor the progress of family planning program regularly at the national and sub-national level, Myanmar has limited surveys, data quality and methodological issues in its Health Management Information System (HMIS), and a scattered rollout of the Logistic Management Information System (LMIS).

**Methods:** To identify viable options for annual monitoring, four data sources: modelled contraceptive prevalence rate for modern methods (mCPR) estimates from Track20’s  Family Planning Estimation Tool (FPET); method-specific prevalence from the  2015-16 Myanmar Demographic and Health Survey (DHS); mCPR estimates and method prevalence from  HMIS and estimates of modern method use (EMU) based on commodity consumption data from LMIS, were used to compare for the years 2015-2017. Estimates of mCPR from HMIS were tested for accuracy based on whether they fell within the 95% confidence interval of mCPR estimates from the FPET for the corresponding years. EMU from LMIS was also tested for those years and states/regions where available.

**Results:** For annual tracking of mCPR, direct estimates of HMIS were considered; they were much higher than those of the DHS survey and were not matched by FPET results, except in Chin and Kayin. To monitor the method mix, HMIS data can be used as these are similar pattern with DHS in both national and State/Regional level except Chin and Kayin. LMIS could be used in annual tracking when there are high reporting rates and valid information of consumption.

**Conclusions:** Track20’s FPET is the method of choice to get valid information for annual monitoring of family planning program.

## Introduction

As family planning is an evidence-based intervention for improving the maternal and newborn health, as well as a cost-effective powerful tool for development, Myanmar committed to the Family Planning (FP) 2020 initiative in 2013 aiming to improve women and children’s health through increased access to the quality family planning services without any disparities ^1, 2^. In order to achieve the objectives of the Myanmar FP 2020 commitment; Contraceptive Prevalence Rate (CPR) must be over 60% by 2020. Myanmar has been endeavoring with strong coordinated efforts among the different sectors: public, private, UN agencies, INGOs, NGOs and donor agencies. Additionally, the family planning program has been implemented under the guidance of the Reproductive Health policy (2002), Five years Reproductive Health Strategic Plans (RHSP) and Costed Implementation Plan for FP 2020 ^1, 2^.

Another driving force for family planning program is high Maternal Mortality Ratio (MMR) in Myanmar. According to UN interagency estimates, the Myanmar maternal mortality ratio (MMR) has reduced from 453 per 100,000 live births in 1990 to 178 per 100,000 live births in 2015; however, this figure was the second highest among ASEAN countries and did not meet the 2015 Millennium Development Goal ^3^. As of the 2014 census, the MMR in Myanmar was 282 deaths per 100,000 live births ^4^ Aiming to reduce maternal morbidity and mortality, family planning service has been accorded as a priority issue in the basic Essential Package of Health Services of Myanmar National Health Plan (2017–21) ^5^.

Myanmar’s family planning programme started in 1991 as a public sector pilot in one township, and then progressively extended to 163 out of 330 townships in 2014. Before 2011, the government had no specific financial allocation for reproductive health commodities, including contraception, and heavily relied on UNFPA supplies. From 2011, the government increased the health budget, allocated a budget for contraceptive commodities and invested more in the family planning program, to allow it to provide more contraceptives, both short- and long-term, free of charge in all public facilities since 2012 ^2^.

Although various inputs have been used in the Myanmar Family Planning program and the contraceptive prevalence rate (CPR) has increased from 41% in 2007 ^6^ to 52.2% in 2016 ^7^, it is estimated to be slightly lower than the target of 60% by 2020 ^5^. At the same time, an unmet need for family planning has been reduced from 19% in 2007 ^6^ to 16% in 2016 ^7^, still falling short of the 2020 target of an unmet need of less than 10% ^5^. Therefore, continuous monitoring plays a role in providing the useful inputs for the directions or strategies of program implementation.

In order to monitor the progress of family planning program, Myanmar uses the FP indicators (CPR, method mixed prevalence, unmet need for family planning and demand satisfaction) mainly from the limited available sources; first ever Demographic and Health Survey (DHS) (2015–16) and Health Management Information System (HMIS), as well as commodity consumption data from Logistic Management Information System (LMIS). HMIS was developed as paper based in 1995 and is now transformed to an electronic system of HMIS as District Health Information System (DHIS) 2. It is the responsibility of Myanmar Ministry of Health and Sports to monitor the performance of all public health facilities while it accounts for local annual census for family planning data and covers both public and private ^8^. In Myanmar, LMIS for reproductive health commodity (RHCLS: Reproductive health commodity logistic system) was initiated as national program in 2016 in selected regions and has been rolled out phase by phase. It provides the information related with stock status as well as commodity consumption data; commodity distributed to the clients, from different public health facilities ^9^. However, systematic tracking of annual family planning progress with valid data source has not yet been set yet.

### Rationale

**Figure 1.**
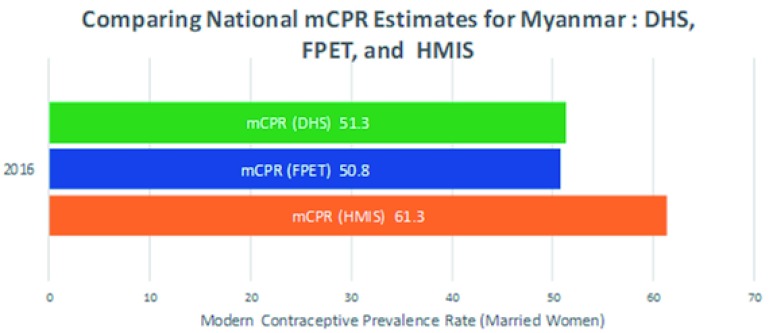
Comparing National mCPR estimates of DHS, FPET, and HMIS.

According to the Myanmar DHS (2015–16), contraceptive use is growing nationally, but there are disparities in use among different states and regions, from the lowest prevalence in Chin State (25%) up to 60% in Yangon and Bago region ^7^. Given the wide variability in contraceptive use and the performance of the FP program by state/region, there is a strong need for valid information about contraceptive use for better annual tracking. Currently, there is limited information on contraceptive use available for the regular monitoring and evaluation as Myanmar has had limited surveys, data quality and methodology issues exist in the Health Management Information System (HMIS), and slow and scattered rollout of the Logistic Management Information System (LMIS) means these service statistics data have limited application for state/regional routine monitoring. The national estimates of mCPR for same period, 2016, are quite different among DHS survey (51.3%) ^7^, result from the Family Planning Estimation Tool (FPET) http://www.track20.org/pages/resources/track20_tools.php; a web application developed by Track 20 project/Avenir Health that uses statistical modeling by incooperating with all available survey and service statistics data to produce annual estimates for key family planning indicators, (50.8%) ^10^ and HMIS (61.3%) ^11^ ( Figure 1). This discrepancy between the different data sources led the program to consider the most reliable data source for both national and subnational annual monitoring on family planning. Also, lack of studies for systematic analysis of the family planning data source called to do this study to set the annual family planning monitoring system in Myanmar.

### Objectives

The objective of this study is to review existing sources of data on the modern contraceptive prevalence rate (mCPR) and method-specific prevalence in Myanmar to identify viable options for annual monitoring of the family planning program at the national and state/regional level.

## Methods

### Data sources

In order to understand what data may best serve annual monitoring of the performance of the family planning program in Myanmar, both at the national and state/regional level, four data sources of modern contraceptive use were used to compare:

1. Modelled mCPR estimates (and confidence intervals) from Track20’s FPET tool (requires free registration), based on nationally and state/regionally representative surveys ^10^ for Myanmar: Myanmar DHS (2015–16), Multiple Indicator Cluster Survey (2010), Fertility and Reproductive Health Survey (1991,1997, 2001 & 2007) (While FPET tool can be incooperated with both survey and service statistics data, FPET run for Myanmar could not use the service statistics data due to data validity.)
2. mCPR and method-specific prevalence from the 2015–16 Myanmar DHS ^7^.
3. mCPR estimates and contraceptive method prevalence from Myanmar’s HMIS system ^11^, based on local annual census conducted by midwives on contraceptive use among married women in their catchment areas; which is different from other routine HMIS indicators and collected once a year at the end of calendar year for the information of contraceptive use including type of methods used, and
4. mCPR from Estimates of modern method use (EMU) tool based on contraceptive commodity consumption data from Myanmar’s LMIS system ^12^ were used to compare ( Table 1). While FPET can be incorporated into service statistics data, it could not be put with the direct estimates of mCPR, other commodity or visit data from service statistics. EMU excel can convert service statistics data to estimated method use (which is a proxy for mCPR) and it can be used as input in the Family Planning Estimation Tool (FPET). EMU tool was developed by Track 20 team and available at its website http://www.track20.org/pages/our_work/innovative_tools/SS_to_EMU_tool.php. Here, EMU tool was used to provide EMU (Estimated method use-a proxy of mCPR) by converting of commodity consumption data to compare with the mCPR from HMIS and FPET tool.

**Table 1.** Data sources for comparison.

Source	mCPR	Years	Method use	Year
FPET	X	2015–2017		
HMIS	X	2015–2017	X	2016
LMIS	Limited regional and time trend availability			
DHS			X	2016

While DHS data are the gold standard for monitoring of FP indicators, it does not provide information for annual monitoring. Between the years of surveys, to monitor the annual progress, Track 20 project proposes a FPET tool which is based on a Bayesian, hierarchical model that fits curves to historical data (all available survey and service statistics). The model fits a logistic growth curve to CPR data for all methods to determine the long term trend in contraceptive use and adds a time-series model with autocorrelation to capture country-specific deviations around the long term trend., Since FPET gives almost the same result as the DHS ( Figure 1), it is the most reliable data source for annual estimates so far.

In addition to FPET, to identify the most valid data source and to test whether the routine service statistics data can be used or not for annual monitoring, contraceptive use data from service statistics (HMIS and LMIS) were used to compare, along with the confidence intervals from FPET for three consecutive years from 2015 to 2017 as FPET data were assumed that the most reliable data source apart from DHS. If the specific data were within the 95% of confidence interval of FPET, it was considered as the accurate data in this study to be used for annual monitoring of family planning.

Firstly, estimates of mCPR from the HMIS at both the national and state/regional level were tested for accuracy based on whether they fell within the 95% confidence interval of mCPR estimates of FPET for the corresponding years. The consistency between two sources was matched for each three years. Then, the method-specific prevalence data for both national and state/regional level from HMIS were compared with DHS data for 2016 only. As the FPET could not provide the method mix data, this data could be compared with DHS. Finally, another service statistic data source; commodity consumption data from LMIS, were used to compare for mCPR along with FPET for its accuracy. The mCPR extracted from estimates of method use (EMU) excel tool; by converting the inputs of commodity consumption data, from LMIS were also tested for those years and in states/regions where the LMIS was available.

### Ethics approval and consent to participate

This paper is a secondary analysis of the four different sources of data: contraceptive prevalence rate from Family Planning Estimation Tool of Track 20, 2015–16 Myanmar Demographic and Health Survey (MDHS), Health Management Information System (HMIS) data of Department of Public Health and Logistic Management Information System (LMIS) data of RH commodities from Maternal Reproductive Health Unit of Department of Public Health. Ethics approval for Myanmar DHS was obtained from the Ethics Review Committee of the Department of Medical Research, Ministry of Health and Sports, Myanmar and the secondary data analysis for this study was done after obtaining the permission from the Department of Public Health, Ministry of Health and Sports, Myanmar.

## Results

### Comparing mCPR from HMIS and FPET

**Figure 2.**
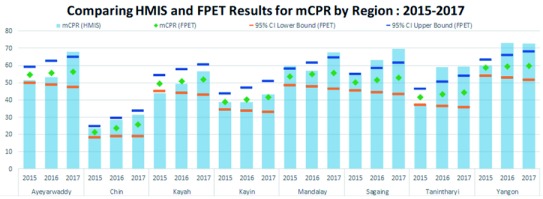
Comparing the HMIS and FPET results of mCPR by regions of at least one year consistent estimates.

**Figure 3.**
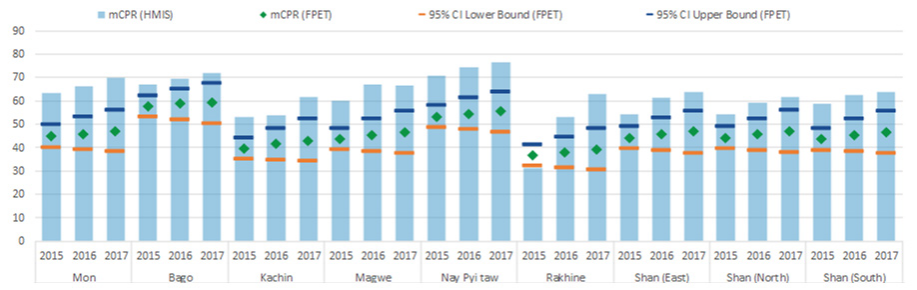
Comparing the HMIS and FPET results of mCPR by regions of zero years consistent estimates.

In comparing HMIS estimates to the point estimates and confidence intervals from FPET, among the total 17 States and Regions, years of consistency for mCPR between these data for three consecutive year could be observed in Figure 2 and Figure 3. The bar shows the HMIS data and FPET data points including upper and lower border (95% CI) were shown on that bar for each three years. Among 17 State/Regions, only two States (Chin and Kayin) produced HMIS-based estimates consistent with FPET results for three consecutive years (e.g. fell within the 95% CI of FPET estimates). Another two (Ayeyarwaddy and Kayah) were consistent with FPET for two of the three years of available HMIS data. Only one year of matching HMIS and FPET results were found in Mandalay, Sagaing, Tanintharyi and Yangon regions ( Figure 2). In the other ten states/regions, estimates of mCPR from HMIS were not within the CI of FPET results for any of the years available ( Figure 3). In general, the HMIS results were most consistent with FPET results in 2015, with six of 17 regions falling within the CI; this dropped to five in 2016 and three in 2017.

**Figure 4.**
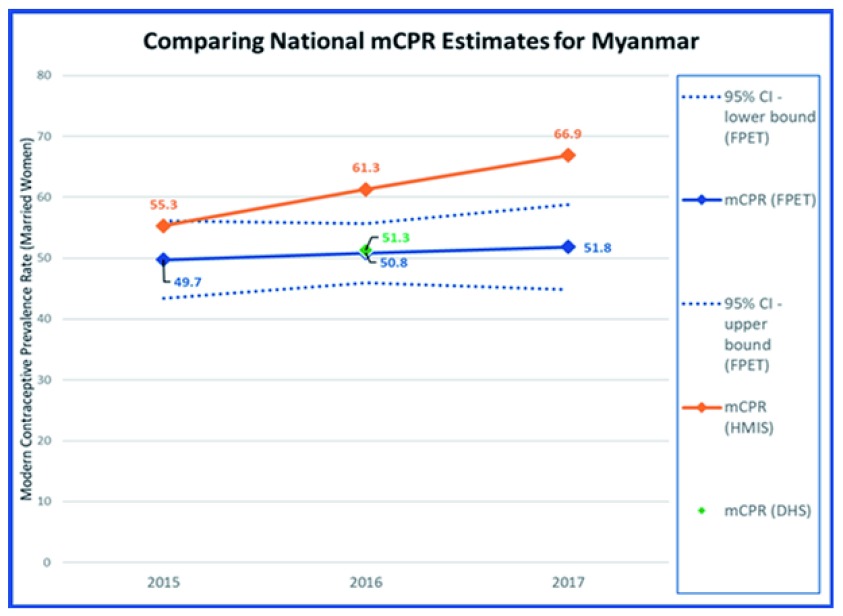
Comparing National mCPR of HMIS and FEPT estimates.

At the national level, only 2015 estimates from HMIS fell within the CI of the FPET mCPR estimates. In general, as at the national level, the HMIS estimates of mCPR appear to over-estimate prevalence when compared to FPET and DHS ( Figure 4).

### Comparing the method prevalence/mix from HMIS and DHS

**Figure 5.**
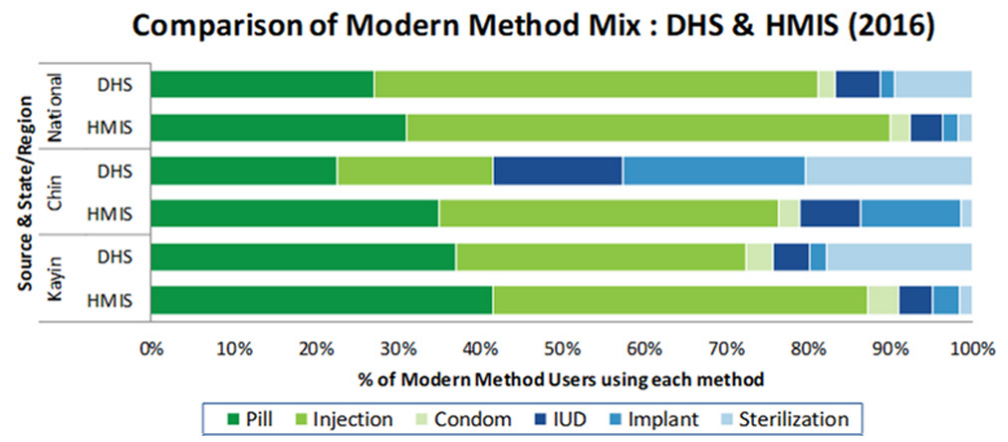
Comparing the modern contraceptive method mix.

In comparing method prevalence, different contraceptive methods (injection, pills, IUD, implants, condom and female sterilization) from HMIS data were compared with the DHS for both national and State/Regional level. Although the mCPR from HMIS data showed consistently higher prevalence compared to the DHS, the same patterns in method mix were observed between the two data at national level, with the exception of female sterilization, which appears to be under-reported in the HMIS system. Across both data sources, injectables were indicated as the most common method in use, making up more than half of all use, followed by pills, used by about a quarter of all married users of modern contraception ( Figure 5).

It was observed that there were similar patterns of method mixes at the national level and most of the 17 states/regions, except Chin and Kayin State. In Chin, rates of use of the long-term methods IUD, implants, and sterilization were considerably higher than the other areas in DHS data. In HMIS data, only IUD and implant were found as higher proportion than that of other State/Regions. Also in Kayin, the higher use of pills than injections was found only in the DHS, while the injection method was the highest proportion in national and other areas in both DHS and HMIS data.

### Comparing the data of LMIS with HMIS data and FEPT results

Regarding estimates of modern method use (EMU, comparable to mCPR) from LMIS commodity consumption data, the LMIS data were found to be quite low in comparison to the HMIS and FPET estimates, except in Southern Shan State. For 2017, in Southern Shan State, while there was a >90% reporting rate, EMU from LMIS data were nearly identical to the mCPR of HMIS (63% vs 64%); however, both values are not within 95% CI of FPET estimates.

## Discussion and conclusion

Access to FP contributes up to a 44% reduction in maternal deaths and a 21% reduction of deaths in children under age 5 ^13^, therefore, Myanmar needs to endeavor towards strong efforts to increase access to family planning services in order to tackle the high MMR issue. In the meantime, setting the systematic approach for continuous monitoring is important to provide the valuable information to be considered for future stratagies.

DHS is the gold standard method of tracking the family planning program; however, annually tracking the national and subnational progress level for equitable access to family planning services is required. When considering the data source for the annual tracking of mCPR, (either estimates from FPET or routine service statistics), the use of direct estimates of HMIS should be considered carefully as it is much higher than the DHS survey and not matched with FPET results except in Chin and Kayin States. It might be due to data quality issue and related with performance of data collectors. Therefore, it should be explored in detail why HMIS shows a high result with 5% annual growth, through reviewing the methodology and validating the data quality.

Currently, there is limited information from documented references about why HMIS overestimates and grows 5% annually, however, in my working experience, there is doubt in the quality of data collected by midwives. According to the methodology, it is necessary to ask all married women under their jurisdiction areas. This is difficult to cover especially in big cities, per-urban as well as hard-to-reach areas. Therefore, it is possible that it includes only the information of women who recieved family planning services from them and is probably higher prevalence than those who could not access health services. However, these are just only the opinions of authors and need to explore systematically and should improve based on that findings.

While the methodology for data collection is quite unusual as a local annual census, it is needed in order to be consistent with other HMIS indicators through the register/record for family planning information and integrated into electronic HMIS (DHIS 2) to get the valid data of family planning service visits and contraceptive users at least for public sectors. Considering the monitoring of the methods mix, as the series of HMIS data are similar pattern with DHS at both national and state/regional level, except Chin and Kayin. Therefore, HMIS data can be used for monitoring of the method mix, except in Chin and Kayin although it is limited in use for tracking of mCPR. The LMIS data could be used for annual tracking of mCPR when there are high reporting rates and valid information of consumption. Thus, LMIS are needed to strengthen in data validity as well as area coverage.

These findings are consistent with other international literature. According to the analysis of three service statistics data (number of: contraceptive commodities distributed to clients, family planning service visits, and current contraceptive users) from 22 countries in Africa and Asia, it was observed that none of these three data were accurate to be used when comparing with United Nations Population Division World Contraceptive Use annual mCPR estimates as the “gold standard.”

Although service statistics data for family planning are insufficient accuracy to use stand-alone like survey data, data on contraceptive commodities distributed to clients, family planning service visits, and current users can be used accurately when combined with survey data in FPET tools to estimates the trends of annual mCPR between the years of surveys ^14^.

### Limitation of study

This study is only secondary data analysis, therefore, the reasons behind the inaccuracy of service statistics data could not explain. It covers only Myanmar data so detail results might be limitation to be applied for other countries context although it is consistent with findings of analysis in 22 countries which is service statistics data were not sufficient in accuracy to be used stand alone for annual tracking.

### Recommendations

In order to get valid information for annual monitoring of Myanmar’s family planning program, Track20’s FPET tool is the method of choice for now. Although service statistics data are insufficient accuracy to use stand-alone like survey data, valid and qualified service statistics data could be used through in-cooperating into FPET tools together with survey data to provide more precise estimation of annual mCPR trend. Therefore, it is important to get valid information of some important service statistic data related with family planning such as family planning service visits, current users and contraceptive commodities distributed to clients.

In future, there is a need to:

- strengthen of HMIS data through reviewing the methodology of data collection for family planning information with further primary research and validating the data quality;
- establish the Family Planning register/record and integrate into electronic HMIS/DHIS 2;
- strengthen the LMIS for reproductive health commodity in terms of data quality as well as area coverage.

## Data availability

### Underlying data

The Demographic and Health Surveys dataset analyzed during the current study ( Myanmar 2015–16) is available in the MEASURE DHS repository ( http://www.measuredhs.com). Access to the dataset requires registration, and is granted to those that wish to use the data for legitimate research purposes. A guide for how to apply for dataset access is available at: https://dhsprogram.com/data/Access-Instructions.cfm.

The HMIS data were requested from the HMIS unit of Department of Public Health, Ministry of Health and Sports, Myanmar, available at https://mm.dhis2.net/hmis/dhis-web-commons/security/login.action. Access to this data is restricted to protect the identities of the subjects; researchers wishing to apply for access should send an email to the Deputy Director General of HMIS, Dr Thet Thet Mu at thetthetmu@mohs.gov.mm, including justification for why access should be granted.

The contraceptive commodity consumption data for LMIS for RH commodities were requested from the Maternal and Reproductive Health Unit of Department of Public Health, Ministry of Health and Sports, Myanmar and John Snow International (JSI); an international organization that are providing technical support for LMIS, also supported in accessing the data. Data can be visualized here. To protect the identities of the subjects, those wishing to gain access to the data should submit an official request to the Director of Maternal and Reproductive Health Division of Ministry of Health and Sports, Myanmar, via ayechum_yi@mm.jsi.com or khaingnwetin@gmail.com, including justification for why access should be granted.
