# Supplementary material for: Setting the stage for strengthened annual monitoring of family planning program performance at the state/national level in Myanmar
Source: Gates Open Res. 2019 Dec 10;3:1541. Originally published 2019 Sep 18. [Version 2] doi: 10.12688/gatesopenres.13012.2 (PMC6913066; doi:10.12688/gatesopenres.13012.2)
Supplement: Peer review history

# Peer review: Reviewer response for version 2

**DOI:** 10.21956/gatesopenres.14246.r28354

**Published:** 2019-12-12

**Authors:** James F. Phillips

**Affiliations:** 1 Heilbrunn Department of Population and Family Health, Mailman School of Public Health, Columbia University, New York City, NY, USA

I have no further comments. The authors have addressed the concerns that I have raised in my review.

I confirm that I have read this submission and believe that I have an appropriate level of expertise to confirm that it is of an acceptable scientific standard.

# Response

**Published:** 2019-12-12

**Authors:** Khaing Nwe Tin

**Affiliations:** Ministry of Health and Sports, Myanmar, Myanmar

Dear Phillips, 

 On behalf of our team, I would like to thank you  for your kind approval for our manuscript. 

 Best regards,

 Khaing

 (corresponding author)

# Peer review: Reviewer response for version 1

**DOI:** 10.21956/gatesopenres.14120.r28054

**Published:** 2019-10-30

**Authors:** James F. Phillips

**Affiliations:** 1 Heilbrunn Department of Population and Family Health, Mailman School of Public Health, Columbia University, New York City, NY, USA

**Overview**

 This manuscript aims to describe progress with family planning trends in Myanmar. Despite a long legacy of isolation of this country from externally supported programs and research, monitoring systems are in place that provide basic information on trends in contraceptive use. Four data sources are marshalled for this investigation, providing a basis for comparative analysis. A Demographic and Health Survey is available, permitting comparison of Myanmar with other countries. Service statistics data are adjusted with modeling tools to permit assessment of trends at the regional level. Results based on service statistics data contrast with survey results, in that levels of contraceptive use are consistently higher than DHS estimates. The analysis is mainly focused on comparison of data sources and results, with results that lead the authors to conclude that a tool known as “Track20 Family Planning Estimation Tool” is the method of choice.

 This manuscript addresses a gap in the regional literature. While much is known about contraceptive use levels and trends in neighboring countries, Myanmar has had relatively little systematic attention. I found the rendition of the history of family planning program development in Myanmar to be helpful, as this information is somewhat difficult to locate elsewhere in the social and demographic literature. Also, the description of data limitations that appears in the Rationale section is useful background information.

**Comments on the Methods section**

 This paper is more focused on the comparison of tools rather than the analysis of family planning, per se.  A tool referred to as FPET and another tool LMIS are compared with DHS data, but the methods section is limited to a discussion of the logic of comparison rather than the logic that underlies each tool. The key objective concerns the need for a credible basis for assessing annual progress with family planning program functioning. It is critical to the paper for the reader to understand how results could be affected by methodological differences in the tools employed. Therefore, it is important for the text to clarify how these assessment tools work, either through citation of explanatory articles or through descriptive analysis of the methodologies that they employ. However, tools are cited in the Rationale section without citations or clarification of how they are similar and how they differ. As a consequence, the reader is left to wonder if the differences portrayed are actually significant. This rather simple addition to the paper would substantially strengthen the presentation. This reviewer recommends application of statistical procedures that compare cross-sectional time series data. By employing a method for statistical comparison, the conclusions and recommendations that are reached would have the credibility that is associated with the comparison of trends with confidence intervals ^1^. 

 Comparisons of method mix suggest that different tools produce similar use profiles but contrasting levels of use with health management information system (HMIS) data generating higher use estimates than corresponding DHS data. Under-estimation of the denominators for HMIS or over-estimation of continuity of use may explain these estimation differences. How such differences could arise is not adequately discussed. 

*The Recommendations section*

 The paper reads as if the different results that are observed are the end of the research process rather than an indication that further research is needed.  Recommendations to strengthen the HMIS through reviewing the methodology is not likely to have much practical meaning. Nor are the other recommendations actionable. Exhortations to strengthen something in general does not convey practical guidance on what the program should actually do.

 This reviewer has not had access to the data, but an illustrative example merits consideration by the authors, either by proposing some other adjustment procedure or by pursuing a version of what I recommend. Consider the following: At one point in the paper, the DHS is alluded to as the “gold standard” for mCPR estimation. The FPET and DHS estimates are similar (Figure 1), but HMIS data appear to be over-estimating the mCPR. If the DHS is considered the gold standard, then a plausible proposition is that its data can be used to develop areal adjustment factors. A regression analysis, with DHS data employed as the dependent variable, HMIS data as an independent variable, with direct and interaction effects for population characteristics and locality indicators could be used to develop regression weights that estimate the discrepancies associated with a set of indicators. The regression weights would define adjustment factors for correcting data to a DHS standard. The correspondence of FPET with the DHS invites an approach that would use the FPET as the “silver standard” for HMIS adjustment. While the paper might not take this up as a technical task, it should include discussion of methods of data adjustment that results appear to invite. This would be a more cogent set of recommendations than the text that now appears in the Recommendations section.

*The figures*

 Figures 2 and 3 are hard to read and needlessly complex to interpret. There are many regions with pronounced variance between regions. Are there take home messages from these data that could be summarized more simply? For example, it is possible that the magnitude of discrepancies is associated with the level of the estimated mCPR. If HMIS level were on the Y axis and FPET were the X axis a simple regression line would simulate correspondence between the data sources, and the scatter around that line would show the pattern of discrepancy by level of mCPR.  

 The title of Figure 3 is confusing. I think that Figure 3 could be dropped. 

 Figure 4 is a largely empty depiction of a few data points. This could be replaced with a table.

 Figure 5 is visually confusing. For some reason, data correspondence in Chin is very different from the other regions. Coloration might help, but tabular data might be more interpretable to a reader.

**Minor comments and suggestions**

 The text of this paper begins with a reference to the maternal mortality rate, but the paper is on a different topic. This reviewer suggests that the authors open the paper with a statement that is a more direct reference to topic of the article and the lack of systematic attention to family planning research in Myanmar and the rationale for the paper. At some point in the discussion, reference to the relationship of family planning with the maternal mortality rate would be informative.

I confirm that I have read this submission and believe that I have an appropriate level of expertise to confirm that it is of an acceptable scientific standard, however I have significant reservations, as outlined above.

## References

[1] : Pooling of Time Series and Cross Section Data. *Econometrica*.1968;36(2) : 10.2307/1907490 10.2307/1907490

# Response

**Published:** 2019-12-03

**Authors:** Khaing Nwe Tin

**Affiliations:** Ministry of Health and Sports, Myanmar, Myanmar

Dear Phillips,

 Thank you so much for your comments and suggestions and I would like to response as follows.

**Comments on the Methods section**

**This paper is more focused on the comparison of tools rather than the analysis of family planning, per se.  A tool referred to as FPET and another tool LMIS are compared with DHS data, but the methods section is limited to a discussion of the logic of comparison rather than the logic that underlies each tool. The key objective concerns the need for a credible basis for assessing annual progress with family planning program functioning. It is critical to the paper for the reader to understand how results could be affected by methodological differences in the tools employed. Therefore, it is important for the text to clarify how these assessment tools works, either through citation of explanatory articles or through descriptive analysis of the methodologies that they employ.** **However, tools are cited in the Rationale section without citations or clarification of how they are similar and how they differ. As a consequence, the reader is left to wonder if the differences portrayed are actually significant. This rather simple addition to the paper would substantially strengthen the presentation. This reviewer recommends application of statistical procedures that compare cross-sectional time series data. By employing a method for statistical comparison, the conclusions and recommendations that are reached would have the credibility that is associated with the comparison of trends with confidence intervals** **.**

 Thank you for your comments and allow me to respond as follows;

 Actually, the main objective of this study is focused to compare the different data source for FP indicators to select the most reliable one for annual monitoring between the years of survey (DHS). As there is no other annual survey in Myanmar, the data from FPET is currently the most reliable source to monitor as it gives the same results as DHS. But, the other routine services like HMIS and LMIS are available, therefore, I would like to test the data whether service statistics data are reliable to use or not for annual monitoring (especially for State and Regional level) and to get the evidence which one is the most viable data sources (FPET/HMIS/LMIS) in Myanmar.

 To be compared, four data sources (DHS, FPET, HMIS and LMIS) were used. (Previously I wrote as “compared” rather than “used” that mislead to readers). For annual progress of mCPR, services statistics data (HMIS and LMIS) were compared along with 95% CI of FPET for their accuracy (because FPET is most reliable source for the years between DHS) for three consecutive years. If the service statistics data points were fell within 95% CI of FPET, it was assumed as accurate and check how many years of consistency between them for each State/Regions (Figure 2 and 3).

 For national comparison of mCPR between HMIS and FPET including 95%CI, three years data points were plotted as trend and DHS was put as one point in 2016 (Figure 3). It also shows the HMIS data were much over estimates of FPET and DHS.

 When HMIS data were compared with FPET, the direct estimates of mCPR from HMIS were used. However, LMIS could not provide the mCPR to compare with FPET. LMIS can provide only commodity consumption data. Therefore, we have to use EMU (Estimated method use) excel tool which is developed by Track 20 team and it can provide EMU (Estimated method use-a proxy of mCPR) by converting commodity consumption data from LMIS.

 The FPET and EMU tools are not comparable and EMU results can be used as input for FPET if we want to incorporate service statistics data into FPET in addition to survey data. But it is not the case of this study as Myanmar FPET estimates use only survey data (due to poor data validity of service statistics data). Here EMU tool was used to provide EMU (Estimated method use-a proxy of mCPR) by converting of commodity consumption data. The links of these tools are also added in revise version.     

  For comparing the pattern of method mix, FPET cannot provide that data, we used DHS data for only that year of survey (2016) and compared with HMIS data. So, actually it doesn’t compare the two tools (FPET and LMIS tool) and compare the service statistics data (HMIS and LMIS through EMU tool) with FPET for mCPR progress and HMIS with DHS for method mix.

 To be more clear, I have tried to edit the methodology section as follows and added text are made as italic;

**Methods**

**Data sources**

 In order to understand what data may best serve annual monitoring of the performance of the family planning program in Myanmar, both at the national and state/regional level, four data sources of modern contraceptive use:

 < >Modelled mCPR estimates (and confidence intervals) from Track20’s FPET, based on nationally and state/regionally representative surveys for Myanmar: *Myanmar DHS (2015-16), Multiple Indicator Cluster Survey (2010), Fertility and Reproductive Health Survey (1991, 1997, 2001 & 2007).* *(7). (While FPET tool can be incooperated with both survey and service statistics data, FPET run for Myanmar could not use the service statistics data due to data validity.)* *mCPR and* Method-specific prevalence from the 2015-16 Myanmar DHS (6).mCPR estimates and contraceptive method prevalence from Myanmar’s HMIS system (8) based on local annual census conducted by midwives on contraceptive use among married women in their catchment areas; *which is different from other routine HMIS indicators and collected once a year at the end of calendar year for the information of contraceptive use including type of methods used*, and mCPR from Estimates of modern method use (EMU) tool based on contraceptive commodity consumption data from Myanmar’s LMIS system (9); were used to compare (Table 1). *(While FPET can be incorporated service statistics data, it could not be put the direct estimates of mCPR, other commodity or visit data from service statistics. EMU excel tool can* *convert service statistics data to Estimated method use (which is a proxy for mCPR) to be can be used as input in the Family Planning Estimation Tool (FPET). EMU tool was developed by Track 20 team and available at its website* *http://www.track20.org/pages/our_work/innovative_tools/SS_to_EMU_tool.php* *.* *Here, EMU tool was used to provide EMU (Estimated method use-a proxy of mCPR) by converting of commodity consumption data.)* *strengthen of HMIS data through reviewing the methodology of data collection for family planning information with further primary research and validating the data quality;* *establish the Family Planning register/record and integrate into electronic HMIS/DHIS;* *strengthen the LMIS for reproductive health commodity in terms of data quality as well as area coverage.*

**Comments**

**This reviewer has not had access to the data, but an illustrative example merits consideration by the authors, either by proposing some other adjustment procedure or by pursuing a version of what I recommend. Consider the following: At one point in the paper, the DHS is alluded to as the “gold standard” for mCPR estimation. The FPET and DHS estimates are similar (Figure 1), but HMIS data appear to be over-estimating the mCPR. If the DHS is considered the gold standard, then a plausible proposition is that its data can be used to develop areal adjustment factors. A regression analysis, with DHS data employed as the dependent variable, HMIS data as an independent variable, with direct and interaction effects for population characteristics and locality indicators could be used to develop regression weights that estimate the discrepancies associated with a set of indicators. The regression weights would define adjustment factors for correcting data to a DHS standard. The correspondence of FPET with the DHS invites an approach that would use the FPET as the “silver standard” for HMIS adjustment. While the paper might not take this up as a technical task, it should include discussion of methods of data adjustment that results appear to invite. This would be a more cogent set of recommendations than the text that now appears in the Recommendations section.**

 Actually, FPET tool is based on Bayesian, hierarchical model that fits curves to historical data (all available survey and service statistics). The model fits a logistic growth curve to CPR data for all methods to determine the long term trend in contraceptive use and adds a time-series model with autocorrelation to capture country-specific deviations around the long term trend.  Since FPET gives almost the same result as DHS, it is assumed as the most reliable data source for annual estimates so far to compare the service statistics data (HMIS and LMIS) in this study. I hope it has already incorporated the adjustment for comparison of HMIS data with DHS. Also, I will add about this in methodology section in my revise version.

**Comments for the figures**

**Figures 2 and 3 are hard to read and needlessly complex to interpret. There are many regions with pronounced variance between regions. Are there take home messages from these data that could be summarized more simply? For example, it is possible that the magnitude of discrepancies is associated with the level of the estimated mCPR. If HMIS level were on the Y axis and FPET were the X axis a simple regression line would simulate correspondence between the data sources, and the scatter around that line would show the pattern of discrepancy by level of mCPR. The title of Figure 3 is confusing. I think that Figure 3 could be dropped.**

****

 In figure 2 and 3, it shows year of consistency for mCPR between the HMIS and FPET estimates including 95%CI for each 17 State/Regions for three consecutive year’s data. The bar shows the HMIS data and FPET data points including upper and lower border (95% CI) are shown on that bar for each three years for 17 State/Regions. We can see the consistency that whether HMIS data (top of the bar) are fell within these upper and lower borders of FPET. For me, it is not easy to draw the regression line for two data sources of three years data points for each 17 State/Regions in one picture. Also, I found that there is no relation between the consistency and magnitude of value. The key messages are there are only two States (Chin and Kayin) for consistency in all three years (Figure 2) and some are two years and some are only one year consistent. About half of State/Regions show no consistency at all (Figure 3) for three years. It means that direct estimates of mCPR from HMIS are not consistency with FPET and not accurate to be used for annual monitoring. I will change the title for Figure 3 as “Comparing the HMIS and FPET results of mCPR by regions of *none of the year* consistent estimates” to be more clear as previously mentioned that “ by regions of no consistent estimates”.

**Comments: Figure 4 is a largely empty depiction of a few data points. This could be replaced with a table.**

 Thank you for your advice. Actually, I would like to show that the comparison of HMIS and FPET (with 95% CI) for national mCPR with trend (three years data points) and rate of progress. It highlights HMIS data were overestimates of FPET for three years (even over the upper border in 2016 and 2017) and DHS as well in 2016. So, I think it could not easy for readers to see these points from table.

**Comments: Figure 5 is visually confusing. For some reason, data correspondence in Chin is very different from the other regions. Coloration might help, but tabular data might be more interpretable to a reader.**

 In Figure 5, it was aimed to show the comparison of different contraceptive method mix (Injection, pills, IUD, implants, condom and female sterilization) prevalence data of HMIS and DHIS (2016) as well as the comparison of national level and state/regional level (Chin and Kayin) where there were different patterns of national level. The key messages are the method mix pattern of HMIS and DHS are quite similar except female sterilization. And while injection was the highest proportion at national level (both HIS and DHS), high proportions of long term methods and female sterilization were found at Chin from DHS (quite different with HMIS) and pills use was the highest in Kayin in DHS. It means that HMIS data were quite similar with DHS for method mix at national and other State/regions except Chin and Kayin. So, I think it might be not easy for readers to interpret if data were shown table.

**Minor comments and suggestions!**

**The text of this paper begins with a reference to the maternal mortality rate, but the paper is on a different topic. This reviewer suggests that the authors open the paper with a statement that is a more direct reference to topic of the article and the lack of systematic attention to family planning research in Myanmar and the rationale for the paper. At some point in the discussion, reference to the relationship of family planning with the maternal mortality rate would be informative.**

 Thank you for your suggestions. I edited the introduction a lot with starting of importance of family planning, about Myanmar FP commitment, Myanmar FP program implementation to achieve FP 2020 commitment and reduce MMR, then how importance of monitoring the progress and current data source. And some text *“Also, lack of studies for systematic analysis of the family planning data source called to do this study to set the annual family planning monitoring system in Myanmar”* has added as the last sentence in rationale.

 In discussion part, I have added the following text at the beginning.

*“Access to FP contributes up to a 44% reduction in maternal deaths and a 21% reduction of deaths in children under age 5 (Ref), therefore, Myanmar needs to endevaour strong efforts to increase access to family planning services to tackle the high MMR issue. In the meantime, setting the systematic approach for continuous monitoring is important to provide the valuable information to be considered for future strategies”*

*(Ref: Starbird E, Norton M, Marcus R Investing in Family Planning: Key to Achieving the Sustainable Development Goals. doi: 10.9745/GHSP-D-15-00374)*

 Also, some editions for another reviewer have been incorporated in my revised version.

 Thanks again and best regards,

 Khaing

# Peer review: Reviewer response for version 1

**DOI:** 10.21956/gatesopenres.14120.r27972

**Published:** 2019-10-24

**Authors:** Janine Barden-O'Fallon

**Affiliations:** 1 Carolina Population Center, Maternal & Child Health Department, Gillings School of Global Public Health, University of North Carolina at Chapel Hill, Chapel Hill, NC, USA

I thank the authors for providing a proof-of-concept from Myanmar on the application of the Family Planning Estimation Tool (FPET) for assessing routine data on key family planning outcomes. The article focuses on the potential for annual assessment of the modern contraceptive prevalence rate (mCPR) and contraceptive method mix (alternately referred to as “method prevalence”), making the justifiable argument that routine monitoring of family planning outcomes is important to the management and success of national family planning programs.

 While the authors find that FPET produces estimates close to DHS estimates (i.e., the gold standard), and that health management information system (HMIS) and logistics management information system (LMIS) estimates do not consistently do so, their recommendations revolve around the strengthening of the HMIS and LMIS. There are many good reasons for this, including that health information systems evolve and system improvements can be ongoing. This means that HMIS and LMIS can become more reliable as data sources over time. Assessing how and why data quality problems arise and implementing practices that can reduce the problems are a big step toward that end. Standardizing the processes for data collection, reporting, analysis and use; implementing feedback systems for course corrections; and building and maintaining the human resources and capacity for managing the systems are also necessary investments to achieve full system functionality.

 My comments and suggestions for minor revisions are provided below.

 Introduction:

- It would be helpful to have contextual information about the development of the HMIS and LMIS in this section.
- Additionally, some information on commodity consumption data may help readers better understand and interpret the findings.

Methods:

- Please name or describe the data sources included in the FPET tool in Myanmar.
- It should be made clear that the FPET and DHS are being used as the standards against which the HMIS and LMIS estimates are being compared. The methods section reads as though these four data sources are being compared against each other, which is not really the case.
- The HMIS mCPR estimate is based on “local annual census conducted by midwives on contraceptive use among married women in their catchment area.”  This seems to be an unusual source of data for an HMIS; can the authors provide more information on this?

Recommendations:

- This section introduces some concepts that are not previously mentioned in the article (“the family planning register,” the “DHIS tool”), thus the basis for the recommendations may not be well-understood by most readers.

Discussion & Conclusion:

- Please see the second sentence in the first paragraph of this section and revise for clarity.
- A tie-in to other literature on this topic is missing; what has other research on this topic found and how does it fit with these results?
- Do authors have any explanation about why HMIS mCPR estimates appear to be over-estimates? What specific “data quality and methodology” issues have been identified? What is being done to improve data quality?
- Information on study limitations is missing.
- “Data” is plural; the document needs editing to ensure proper noun-verb agreement.

I confirm that I have read this submission and believe that I have an appropriate level of expertise to confirm that it is of an acceptable scientific standard.

# Response

**Published:** 2019-10-28

**Authors:** Khaing Nwe Tin

**Affiliations:** Ministry of Health and Sports, Myanmar, Myanmar

Dear Sir,

Thank you so much for your comments and these are very useful to revise my article. I agreed all of your points and would like to respond as follows;

Introduction:

- It would be helpful to have contextual information about the development of the HMIS and LMIS in this section.
- Additionally, some information on commodity consumption data may help readers better understand and interpret the findings.

I will add one paragraph in the last of introduction section regarding the current data source for FP monitoring in Myanmar and will also explain about the HMIS and LMIS including commodity consumption data as follows:

*“In order to monitor the progress of family planning program, Myanmar uses the FP indicators (CPR, method mixed prevalence, unmet need for family planning and demand satisfaction) from the limited available sources mainly; first ever Demographic and Health Survey (DHS)(2015-16) and Health Management Information System (HMIS), as well as commodity consumption data from Logistic Management Information System (LMIS). HMIS was developed since 1995 as paper based and now transformed to electronic system of HMIS as District Health Information System (DHIS) 2. It is the health information system of Myanmar Ministry of Health and Sports to monitor the performance of all public health facilities while it accounts as local annual census for family planning data and covers both public and private. In Myanmar, LMIS for reproductive health commodity (RHCLS: Reproductive health commodity logistic system) is initiated as national program since 2016 in selected region and rolling out phase by phase. It provides the information related with stock status as well as commodity consumption data; commodity distributed to the clients, from different public health facilities. However, systematic tracking of annual family planning progress with valid data source has not yet been set yet.”*

**Methods: **

- Please name or describe the data sources included in the FPET tool in Myanmar.

I will insert the names of surveys that included in FPET for Myanmar as follows in data source part:

“Modelled mCPR estimates (and confidence intervals) from Track20’s FPET, based on nationally and state/regionally representative surveys (7): *Myanmar DHS (2015-16), Multiple Indicator Cluster Survey (2010), Fertility and Reproductive Health Survey (1991, 1997, 2001 & 2007).*

- It should be made clear that the FPET and DHS are being used as the standards against which the HMIS and LMIS estimates are being compared. The methods section reads as though these four data sources are being compared against each other, which is not really the case.

Thanks for suggestion and I removed the clause of “four data sources were compared” and edited as follows:

*“In addition to FPET, to identify the most valid data source for annual monitoring, service statistics data (HMIS and LMIS) of CPR were compared along with the confidence intervals from FPET for three consecutive years from 2015 to 2017.”*

- The HMIS mCPR estimate is based on “local annual census conducted by midwives on contraceptive use among married women in their catchment area.”  This seems to be an unusual source of data for an HMIS; can the authors provide more information on this?

Yes, it is unusual data collection and I also added some information in introduction and added in data source as follows;

“mCPR estimates and contraceptive method prevalence from Myanmar’s HMIS system (8), based on local annual census conducted by midwives on contraceptive use among married women in their catchment areas; *which is different from other routine HMIS indicators and collected once a year at the end of calendar year for the information of contraceptive use including type of methods used,*”

**Recommendations: **

- This section introduces some concepts that are not previously mentioned in the article (“the family planning register,” the “DHIS tool”), thus the basis for the recommendations may not be well-understood by most readers.

I have added the following paragraph in discussion to be more understandable that recommendation.

*“While the methodology for data collection is quite unusual as local annual census, it is also needed to reconsider to be consistent with other HMIS indicators through the register/record for family planning information and integrated into electronic HMIS (DHIS 2) to get the valid data of family planning service visit and contraceptive users at least for public sectors.”*

**Discussion & Conclusion: **

- Please see the second sentence in the first paragraph of this section and revise for clarity.

I have revised as follows;

*When considering the data source for the annual tracking of mCPR (either estimates from FPET or routine service statistics), the use of direct estimates of HMIS should be considered carefully as it is much higher than the DHS survey and not matched with FPET results except in Chin and Kayin States.*

- A tie-in to other literature on this topic is missing; what has other research on this topic found and how does it fit with these results?

I have added the findings of international literature as follows;

*These findings are consistent with other international literature. According analysis of three service statistics data (number of: contraceptive commodities distributed to clients, family planning service visits, and current contraceptive users) from 22 countries in Africa and Asia, it was observed that none of these three data were accurate to be used when comparing with United Nations Population Division World Contraceptive Use annual mCPR estimates as the "gold standard."*

*Although service statistics data for family planning are insufficient accuracy to use stand-alone like survey data, data on contraceptive commodities distributed to clients, family planning service visits, and current users can be used accurately when combined with survey data in FPET tools to estimates the trends of annual mCPR in the years between surveys.*

*(Ref)* Magnani RJ, Ross J, Williamson J, Weinberger M. Can family planning service statistics be used to track population-level outcomes?.Glob Health Sci Pract. 2018;6(1):93-102.https://doi.org/10.9745/GHSP-D-17-00341

- Do authors have any explanation about why HMIS mCPR estimates appear to be over-estimates? What specific “data quality and methodology” issues have been identified? What is being done to improve data quality?

As there has not been explored systematically yet, I have not known the exact reasons as this is just secondary data analysis, so I put in the recommendation. But I would add as follows in discussion to be more clear to readers;

*Currently, it has limited information from documented references about that why HMIS appear overestimates and grow 5% annually, however, to my working experiences, it is doubtful in data quality by midwives. According to the methodology, it is needed to ask all married women under their jurisdiction areas, it is difficult to cover especially in big city, per-urban as well as hard-to-reach areas. Therefore, it might be possible that it includes only the information of women those who got family planning services from them and probably higher prevalence than those who could not access the health services. However, these are just only the opinions of authors and need to explore systematically and should improve based on that findings.*

- Information on study limitations is missing.

Thank you for your good point to remind me. I missed it and now I will add as follows;

*“This study is only the secondary data analysis, therefore, the reasons behind the inaccuracy of service statistics data could not explain. It covers only Myanmar data so detail results might be limitation to be applied for other countries context although it is consistent with findings of analysis in 22 countries which is service statistics data were not sufficient in accuracy to be used stand alone for annual tracking.”*

 

- “Data” is plural; the document needs editing to ensure proper noun-verb agreement.

Thank you for your suggestions and will edit accordingly.

Best regards,

Corresponding Author: Khaing Nwe Tin
